# A More Efficient and Safer Improved Percutaneous Pedicle Screw Insertion Technique—Trajectory Dynamic Adjustment Technique, Technical Note, and Clinical Efficacy

**DOI:** 10.1111/os.14260

**Published:** 2024-10-15

**Authors:** Hao Li, Zhiguo Ding, Bin Wei, Zhihao Ma, Jing Xie, Yonghao Tian, Lianlei Wang, Xinyu Liu, Suomao Yuan

**Affiliations:** ^1^ Department of Orthopedics Beijing Jishuitan Hospital, Capital Medical University Beijing China; ^2^ Department of Orthopedics Qilu Hospital of Shandong University Jinan Shandong People's Republic of China; ^3^ Department of Orthopedics Shouguang People's Hospital Weifang Shandong People's Republic of China; ^4^ Shandong Provincial Maternal and Child Health Care Hospital Affiliated to Qingdao University Jinan China; ^5^ Department of Dermatology The Second Affiliated Hospital of Xi'an Jiaotong University Xi'an Shanxi People's Republic of China

**Keywords:** facet joint violation, lumbar, minimally invasive, percutaneous pedicle screw fixation, radiation exposure

## Abstract

**Objective:**

Percutaneous pedicle screw fixation (PPSF) technique requires a very precise entry point of the Jamshidi needle, which leads to repeated adjustments, damaging the pedicle and increasing radiation exposure. This study was designed to propose an improved percutaneous pedicle screw fixation technique‐trajectory dynamic adjustment (TDA) technique, and evaluate its feasibility and assess the clinical outcomes.

**Method:**

A total of 445 patients with lumbar spondylolisthesis or lumbar spinal stenosis associated with instability from June 2017 to May 2022 were included in the retrospective study. They were randomly separated into two groups. Two hundred thirty‐one patients underwent TDA technique (TDA group). Two hundred fourteen patients underwent traditional PPSF technique (PPSF group). All patients underwent postoperative CT to assess the accuracy of screw placement, superior facet joint violation (FJV). The evaluated clinical outcomes were needle insertion time, radiation exposure, blood loss, hospital stay, the Japanese Orthopedic Association (JOA) score, the Visual Analogue Scale (VAS) scores for lower back pain (LBP), and leg pain, lumbar interbody fusion rate, and postoperative complications. The independent‐sample *t* test and paired t‐test were used for continuous data. The contingency table and Mann–Whitney *U* test were used for categorical data.

**Results:**

The time of the insertion in TDA group was significantly lower than that in PPSF group (*p* < 0.05). Similarly, the fluoroscopy frequency in TDA group was significantly lower than that in PPSF group (*p* < 0.05). There was no difference in intraoperative blood loss and hospital stay between the two groups (*p* > 0.05). Overall, there was no significant difference in the proportion of clinically acceptable screws between the two groups (*p* > 0.05). In addition, the lateral screw misplacement in TDA group was higher. Moreover, FJV rate was significantly lower than that in PPSF group (*p* < 0.05). In both TDA group and PPSF group, postoperative back and leg pain and the JOA score were significantly improved (*p* < 0.05). However, there were no significant differences in the pre‐ and postoperative VAS score for back and leg pain and the JOA score, JOA recovery rate, intervertebral fusion rate, and complications rate between the two groups (*p* > 0.05).

**Conclusion:**

Compared to traditional PPSF technique, TDA technique is a safer and more effective procedure which has shorter surgical time, lower radiation exposure, and lower facet joint violation rate.

## Graphical abstract

The present study proposed an improved percutaneous pedicle screw fixation (PPSF) technique‐trajectory dynamic adjustment (TDA) technique. The entry point of this modified technique is slightly deviated outward by 3–5 mm, positioning directly toward the root of the transverse process, and in a relatively horizontal manner, which allows for better anchoring. The purpose of this study was to evaluate the feasibility and advantages of the TDA technique in spinal surgery and verify the safety and effectiveness of this technique.

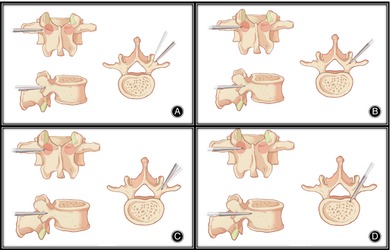

## 1 Introduction

In 1982, Magerl [1] first reported the use of percutaneous pedicle screw fixation (PPSF) technique in thoracolumbar spine, which had the advantages of small incision, less blood loss and minimal damage to the muscles in the lower back [2]. Since then, percutaneous pedicle screw fixation has been gradually applied in clinical practice. Foley et al. [3] reported the minimally invasive transforaminal lumbar interbody fusion (MIS‐TLIF), which introduced the first generation of percutaneous pedicle screw systems and gradually became the standard instrument used worldwide. With the development of percutaneous pedicle screw technique, it has been applied in minimally invasive surgeries for long segments ranging from the thoracic spine to the pelvis, including various diseases such as spine fracture, intervertebral discitis, scoliosis, and spinal metastases [4, 5, 6].

Compared with the conventional free‐hand technique in open surgery, percutaneous pedicle screw placement not only has the advantage of less injury, but also has been proved to have higher screw placement accuracy [7, 8]. At present, PPSF technique only requires a standard spine table and a C‐arm with high resolution, and without the need for other special equipment, making it suitable for implementation in primary hospitals. The hyperplasia of the facet joint is common in the degenerative spine [9]. Open surgery usually requires adequate exposure to determine the screw entry point. However, severe hypertrophic osteophytes often lead to disrupted anatomic structures, making it difficult to identify, affecting the accuracy of screw placement. PPSF technique is not significantly affected by these hypertrophic osteophytes. In addition, when degenerative scoliosis occurs, the vertebral body often rotates to a certain extent [10]. Severe vertebral rotation during open surgery often affects the surgeon's judgment of the screw placement angle, impacting the accuracy of screw placement. However, PPSF technique can effectively eliminate the effect of vertebral rotation through the adjustment of fluoroscopic angle.

**FIGURE 1:**
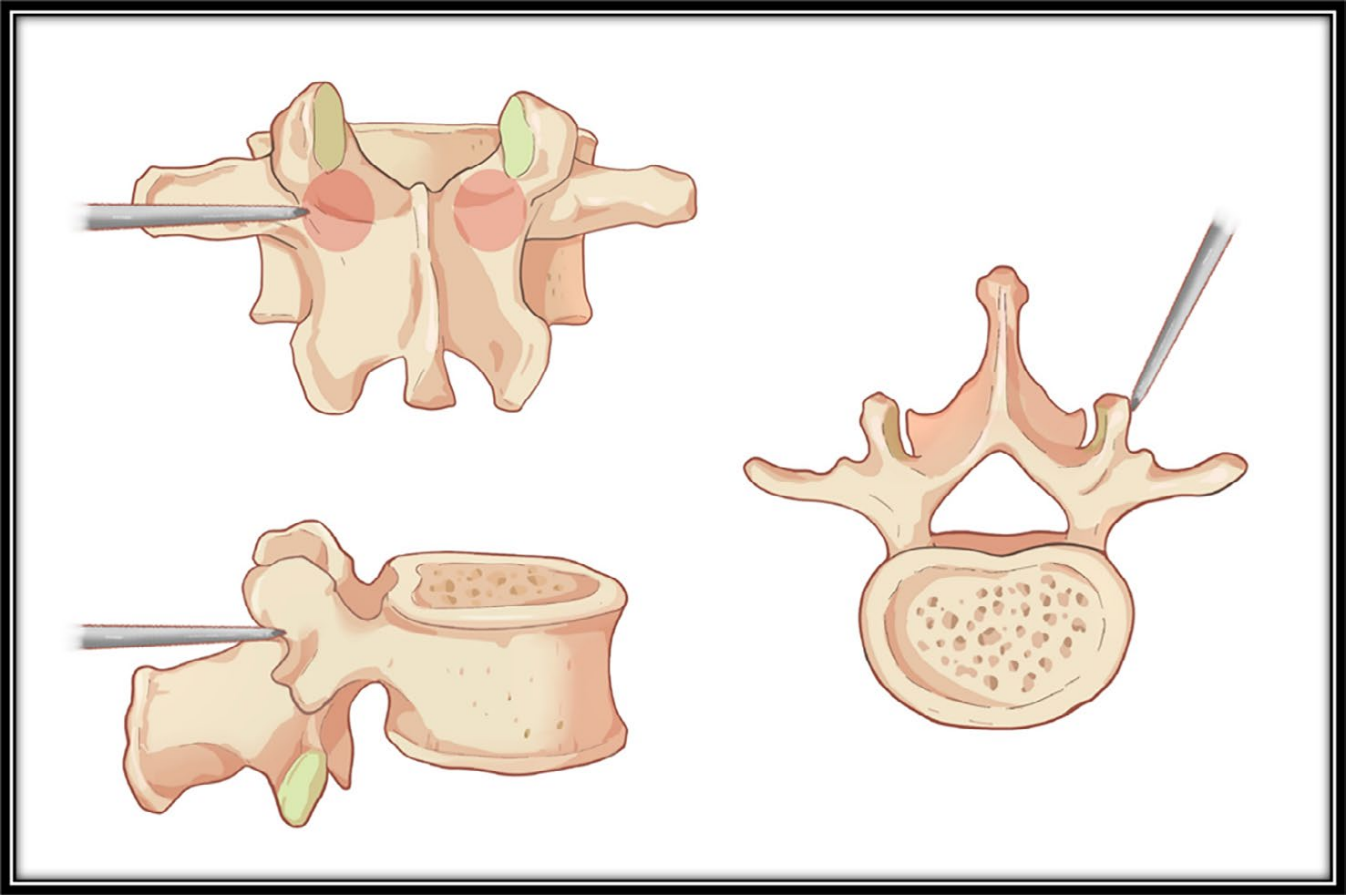
Entry point of traditional percutaneous pedicle screw fixation.

Mobbs et al. [11] gave a detailed and accurate description of the PPSF, which can be divided into three steps. The first step involves the insertion of the Jamshidi needle from the outer border of the pedicle projection into the inner wall of the pedicle under fluoroscopic guidance. The second step is to confirm the tip of the Jamshidi needle has entered the vertebral body under lateral fluoroscopic guidance, allowing the needle to be advanced further. The final step is the placement of the guide wire. However, the classic PPSF technique requires the entry point of the needle to be at “3 or 9 o‐clock” position on the outer border of the pedicle, which corresponds to an oblique plane of the transverse process or accessory process (Figure 1). As a result, the Jamshidi needle is relatively difficult to fix and tends to slide during the actual procedure. Achieving perfect initial anchoring at the 3 o'clock or 9 o'clock positions requires repeated adjustments, which can cause damage to the pedicle and increase the surgical difficulty. This not only affects efficiency but also increases radiation exposure for both the surgeon and the patient.

In the present study, we proposed an improved percutaneous pedicle screw technique—trajectory dynamic adjustment (TDA) technique. The entry point of this modified technique is slightly deviated outward by 3–5 mm, positioning directly toward the root of the transverse process, and in a relatively horizontal manner, which allows for better anchoring. The purpose of this study was to evaluate the feasibility and advantages of the TDA technique in spinal surgery and verify the safety and effectiveness of this technique.

## 2 Materials and Methods

### 2.1 General Patient Information

The institutional review board of the local hospital approved this study (KYLL‐2021(KS)‐055). A total of 445 patients with lumbar spondylolisthesis who received MIS‐TLIF surgery in our hospital from June 2017 to May 2022 were included, including 276 females and 169 males, aged 38–86 year. The inclusive and exclusive criteria were the same for TDA group and PPSF group. Inclusion criteria: Patients with lumbar spondylolisthesis or lumbar spinal stenosis associated with instability (slip distance > 5 mm or range of motion > 10° on flexion and extension radiographs) undergoing MIS‐TLIF. Exclusion criteria: (1) a history of lumbar surgery; (2) the slippage is more than degree 2; (3) accompanied by rheumatoid arthritis, tumors, trauma, or infection.

### 2.2 Study Group

The patients were randomly divided into two groups. The patients in TDA group underwent TDA technique and patients in PPSF group underwent traditional PPSF. For TDA group, there were 231 patients (89 males and 142 females, with an average age of 58.4 years). Eighty four patients were diagnosed with lumbar isthmic spondylolisthesis, 132 patients were degenerative spondylolisthesis, and 15 patients had lumbar spinal stenosis with instability. Among them, 168 patients underwent single‐segment surgery, 46 patients underwent double‐segment surgery, and 17 patients underwent three‐segment surgery. For PPSF group, there were 214 patients (80 males and 134 females, with an average age of 57.5 years). Eighty five patients had lumbar isthmic spondylolisthesis, 116 patients had degenerative spondylolisthesis, and 13 patients had lumbar spinal stenosis with instability. Among them, 154 patients underwent single‐segment surgery, 44 patients underwent double‐segment surgery, and 16 patients underwent three‐segment surgery. The demographics and characteristics of the patients are shown in Table 1.

**TABLE 1:** The demographic s and characteristics of the patients.

		Group A	Group B	*p*
Number of cases (*n*)		231	214	—
Age (year)		58.4 ± 8.0	57.5 ± 8.7	*p* = 0.859
Sex				
Male		89	80	*χ* ^2^ = 0.062 *p* = 0.804
Female		142	134	
Diagnosis				
Lumbar isthmic spondylolisthesis		84	85	*χ* ^2^ = 0.035 *p* = 0.766
Degenerative spondylolisthesis		132	116	
Lumbar spinal stenosis with instability		15	13	
Operative level				
Single‐segment	L1–2	1	0	*χ* ^2^ = 0.034 *p* = 0.983
	L2–3	1	2	
	L3–4	15	14	
	L4–5	119	108	
	L5–S1	32	30	
Double‐segment	L2–4	2	3	
	L3–5	33	31	
	L4–S1	11	10	
Three‐segment	L3–S1	11	9	
	L2–5	6	7	
Meyerding classification				
Grade I		223	217	*χ* ^2^ = 1.17 *p* = 0.557
Grade II		81	65	
Grade III		7	8	

### 2.3 Trajectory Dynamic Adjustment Technique

Compared to the traditional percutaneous pedicle screw fixation technique, there are two key points in this improved trajectory dynamic adjustment technique. First, the initial entry point is shifted outward by 3–5 mm compared to the traditional technique. This can avoid needle tip slippage caused by the steep bone surface of the superior articular process, and allows for better initial anchoring at the more horizontal transverse process base. Typically, this entry point is located on the outer side of the lateral edge of the pedicle, so in the initial stage of needle advancement, it is necessary to increase the inclination angle of the needle (coronal/axial adjustment), so that the needle tip quickly reaches the inner side of the lateral edge of the pedicle. At this point, the inclination angle of the needle can be reduced, and as the needle advances, the needle tip gradually reaches the inner edge of the pedicle. Second, this technique does not strictly require the initial entry point to be located centrally between the upper and lower edges of the pedicle, such as the 3 o'clock or 9 o'clock directions. The initial entry point only needs to be between the upper and lower wall of the pedicle, and during the process of needle advancement, the needle head tilt/tail tilt angle (sagittal adjustment) can be adjusted to gradually reach the central position of the pedicle. This technique does not require high precision for the initial entry point, and the entry point does not have to be located on the midline of the pedicle, nor does it have to beon the inner side of the pedicle outer wall. During the needle advancement process, it can be adjusted according to the position of the initial anchor point, using the trajectory dynamic adjustment technique, which includes coronal/axial trajectory adjustment and sagittal trajectory adjustment. The coronal/axial trajectory adjustment can smoothly guide the entry point, which was originally not on the inner side of the pedicle outer wall, into the pedicle without perforating the inner wall of the pedicle. The sagittal trajectory adjustment can gradually adjust the entry point, which was originally not on the midline of the pedicle, to a position close to the midline.

**FIGURE 2:**
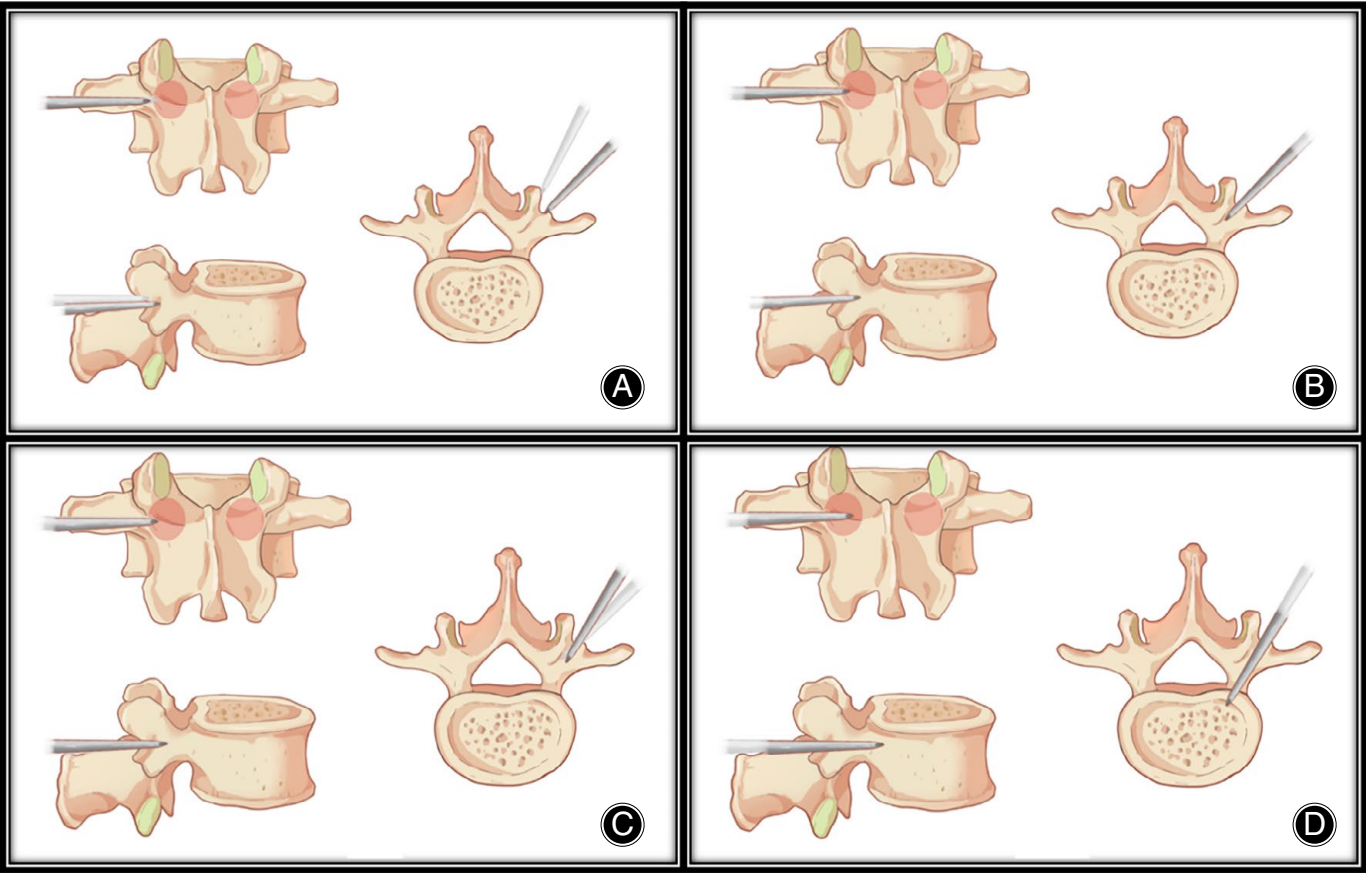
The process of coronal/axial trajectory adjustment. (A) The entry point is more outward than a traditional PPSF, and lateral tilt angle of the needle can be appropriately increased. (B) The needle tip enters the pedicle. (C) Reduce the lateral tilt angle of the needle. (D) The needle tip enters the vertebral body.

An example of coronal/axial trajectory adjustment: When the initial anchoring point is located beyond 5 mm outside the lateral edge of the pedicle, and it is difficult to adjust the entry point inward due to steep bone surface caused by facet joint hypertrophy, the lateral tilt angle of the needle can be appropriately increased, so that the needle can advance more horizontally, and the needle tip can quickly reach the inner side of the outer wall of the pedicle. Once the fluoroscopy confirms that the needle tip has entered the pedicle, the lateral tilt angle of the needle can be reduced, and the needle can be further advanced by hammering until the tip reaches the inner wall of the pedicle (Figure 2).

**FIGURE 3:**
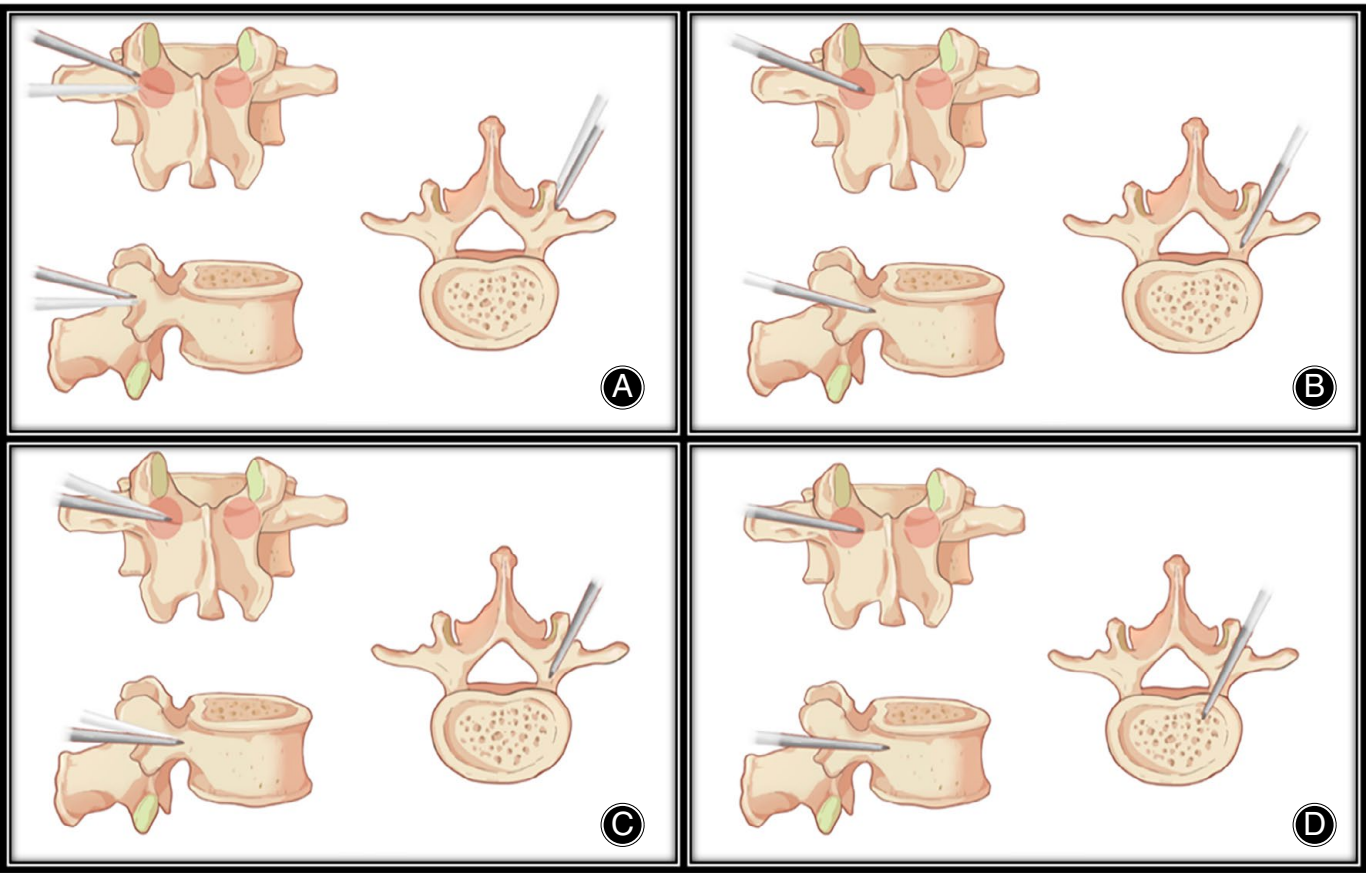
The process of sagittal trajectory adjustment. (A) The entry point is outside and above compared with traditional PPSF, the head tilt angle of the needle should be appropriately increased. (B) The needle tip gradually approached to the midline of the pedicle. (C) Reduce the head tilt angle of the needle. (D) The needle tip enters the vertebral body.

An example of sagittal trajectory adjustment: When the initial anchoring point is outside and above 9 o'clock position, the head tilt angle of the needle should be appropriately increased, so that the needle tip gradually approaches to the midline of the pedicle with the advancement of the Jamshidi needle. After the Jamshidi needle advances 5–10 mm, confirm the needle tip reaches the midline of the pedicle under fluoroscopy. At this point, the head tilt angle of the needle is reduced and the needle continues to advance 5–10 mm, gradually reaching the inner wall of the pedicle (Figure 3).

### 2.4 Radiographic Assessment

The degree of lumbar spondylolisthesis was assessed using the Meyerding Classification System [12]: the degree of spondylolisthesis was 0%–25% as degree I, 25%–50% as degree II, 50%–75% as degree III, 75%–100% as degree IV, and greater than 100% as degree V. All patients underwent postoperative CT to assess the accuracy of screw placement and superior facet joint violation (FJV). The pedicle screw positions were evaluated using the following grade [13, 14]: grade A, screw completely within the pedicle; grade B, pedicle cortical breach of < 2 mm; grade C, pedicle cortical breach of ≥ 2 to < 4 mm; grade D, pedicle cortical breach of ≥ 4 mm. Grade A and B screw positions were considered clinically acceptable, while grade C and D indicated misplacement. Postoperative superior facet joint violation was assessed according to Babu et al. [15], and the degree of violation was classified into four grades: Grade 0, screw not in facet; Grade 1, screw in lateral facet but not in facet articulation; Grade 2, penetration of facet articulation by screw; Grade 3, screw travels within facet articulation.

### 2.5 Clinical Outcome Measures

JOA score, JOA recovery rate, VAS score of low back pain and leg pain, and interbody fusion rate were used to evaluate clinical efficacy [16]. The JOA recovery rate was calculated as follows: recovery rate = (follow‐up score‐preoperative score)/(29‐preoperative score) × 100%. A 10‐cm VAS was used to assess low back pain and leg pain (0 indicated no pain/numbness, and 10 represented the worst pain/numbness). The VAS score was recorded independently by the patients after being given brief instructions by the surgeons. For patients with bilateral leg symptoms, the more severe side was evaluated. All patients underwent at least 2‐year follow‐up after the surgery. At the end of the follow‐up period, they received the appropriate questionnaire by phone. The Brantigan score (0–4 points) for CT scanning was used to evaluate intervertebral fusion [17]. Patients with a score of > 3 were considered fused.

The time from the beginning of the needle insertion to the end of the needle insertion was accurately recorded, and the radiation exposure was assessed by the number of intraoperative fluoroscopies. All screw placement is performed by two senior surgeons, with surgeon Xinyu Liu using traditional technique and surgeon Suomao Yuan using TDA technique.

### 2.6 Statistical Analysis

Statistical analyses were performed using SPSS version 25.0 (IBM Corp., Armonk, NY, USA). All continuous data is expressed as mean ± standard deviation. Differences between the two groups were determined by the independent‐sample *t*‐test. Paired t‐test was used to compare the pre‐ and postoperative data within the group. Contingency table was used to analyze the categorical data, and Mann–Whitney *U* test was used for the data that did not meet the standard normal distribution. The significance level was set to be 0.05.

## 3 Results

### 3.1 Demographic Data

**FIGURE 4:**
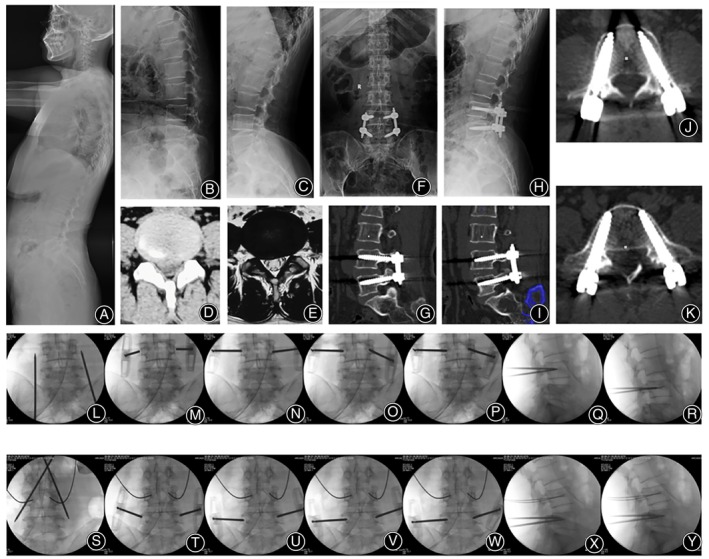
A 65‐year‐old female patient was diagnosed with degenerative spondylolisthesis, and MIS‐TLIF with TDA technique was performed. (A–C) Preoperative x‐rays showed instability of L4‐5. CT (D) and MRI (E) showed disc herniation. (F–K) Postoperative x‐rays and CT showed accurate screw placement. Figure (L) shows the localization of the L4 puncture points. The initial anchoring point on both sides was located 3–5 mm outside the lateral wall of the pedicle, with a slightly smaller angle of puncture needles on both sides (M). After increasing the abduction angle on the left side, the needle tip advanced to the medial wall of the pedicle, which was the ideal path for the screw; meanwhile, the puncture point on the right side slid down to the lower margin of the pedicle, representing a non‐ideal path (N). The left puncture needle maintained its direction and advanced to the inner wall of the pedicle, while the right puncture needle tilted caudally, directing the needle tip toward the cephalad and gradually advancing to the center of the upper and lower margins of the pedicle (O). The right puncture needle continued to advance until the needle tip reached the inner wall of the pedicle (P). Lateral fluoroscopy showed that the needle tips had entered the vertebral body (Q). The needle cores were removed, and guide wires were placed (R). Figure S illustrates the localization of the L5 puncture points. The initial anchoring point on the left side was ideal, while the right side was deviated toward the head side, with a smaller abduction angle (T). When advancing the puncture needle on the left side, it slid caudally to a non‐ideal position, while the needle of right side continued to advance with an increased abduction angle and head tilt angle (U). The left puncture needle tilted caudally, directing the needle tip to the center of the upper and lower margins of the pedicle, and continued to advance; the right side maintained the original angle while continuing to advance (V). Needles of both sides continued to advance until the needle tips reached the inner wall of the pedicle (W). At this point, lateral fluoroscopy showed that the needle tips had entered the vertebral body (X). The needle cores were removed, and guide wires were placed (Y).

**FIGURE 5:**
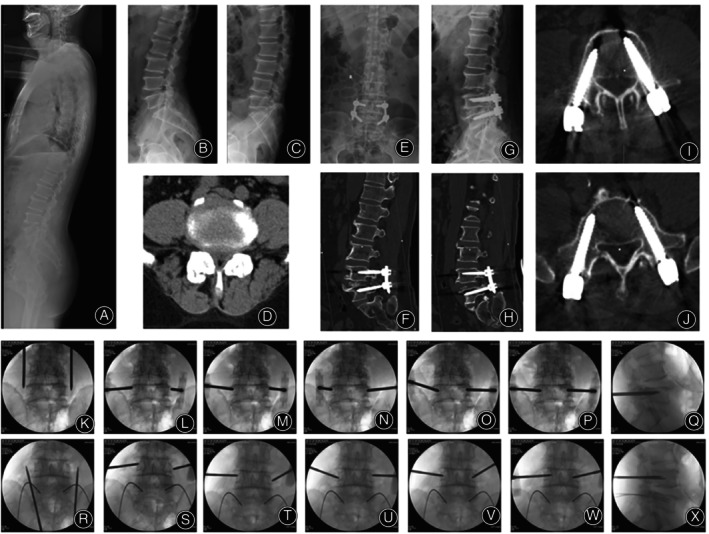
A 60‐year‐old male patient was diagnosed with lumbar spinal stenosis associated with instability, and MIS‐TLIF with TDA technique was performed. (A–C) Preoperative x‐rays showed instability of L4‐5. CT (D) showed lumbar stenosis. (E–J) Postoperative x‐rays and CT showed accurate screw placement. Figure (K) illustrated the positioning of the L5 puncture point. The initial anchoring point on the left side was in a less‐than‐ideal position, leaning toward the head; the initial anchoring point on the right side was slightly external (L). The position of the left anchoring point was not adjusted, with a moderate increase in the head tilt direction, directing the needle tip toward the center of the upper and lower edges of the pedicle; the right anchoring point was adjusted inward, remaining in a less than ideal position, also leaning toward the head side, with a smaller abduction angle (M). The lateral abduction angle was increased, and the head tilt angle was also increased, allowing the needle tip to reach the inner wall of the pedicle; this operation caused the skin to become overly tight, resulting in a smaller abduction angle of the left puncture needle (N). At this point, the right puncture needle had achieved a secure anchoring, prompting an increase in the left puncture needle's abduction angle to continue advancing the needle (O). Slight adjustments were made to both sides of the puncture needles, and the advancement continued until the needle tip reached the inner wall of the pedicle (P). At this moment, the lateral view showed that the needle tips had entered the vertebral body (Q). Figure R illustrated the positioning of the L4 puncture point. The initial anchoring point on the left side leaned toward the head, while the right side leaned toward the tail (S). During the adjustment of the anchoring points, the left side leaned toward the tail, and the right side leaned toward the head (T and U). During the advancement of the needle, the left side increased its tail tilt, while the right side increased its head tilt, allowing the needle tip to reach the inner wall of the pedicle (U and W). At this point, the lateral view showed that the needle tips had entered the vertebral body (X).

In total, 445 patients were included in the present study. For TDA group, there were 231 patients with an average age of 58.4 years (89 males and 142 females). For PPSF group, there were 214 patients with an average age of 57.5 years (80 males and 134 females). There was no difference in terms of demographic presentation between the two groups (*p* > 0.05) (Table 1). An example of TDA technique was shown in Figures 4 and 5.

### 3.2 Perioperative Observational Index

The time from the beginning to the end of the needle insertion in TDA group was 24.4 ± 10.6 min, which was significantly lower than the time of 29.3 ± 11.1 min in PPSF group (*p* < 0.05). Among them, the time of single‐segment, double‐segment, and three‐segment in TDA group was 22.3 ± 6.5, 28.7 ± 9.3 and 33.5 ± 12.4, respectively, and that in PPSF group was 26.4 ± 7.8, 34.7 ± 9.3 and 41.5 ± 12.5, respectively, and the difference was statistically significant (*p* < 0.05). Similarly, the fluoroscopy frequency in TDA group was 30.2 ± 14.3 times, which was significantly lower than 38.2 ± 15.6 times in PPSF group (*p* < 0.05). And the fluoroscopy frequency of single‐segment, double‐segment, and three‐segment in TDA group was 26.3 ± 7.5, 37.7 ± 8.5, 48.7 ± 16.2, respectively, and that in PPSF group was 33.4 ± 8.3, 46.7 ± 10.3, and 60.2 ± 19.5, respectively, and the difference was statistically significant (*p* < 0.05). There was no difference in intraoperative blood loss and hospital stay between the two groups (*p* > 0.05) (Table 2).

**TABLE 2:** Perioperative outcomes of patients in the two groups.

		Group A	Group B	*p*
Time of needle insertion (min)	Single‐segment	22.3 ± 6.5	26.4 ± 7.8	0.001
	Double‐segment	28.7 ± 9.3	34.7 ± 9.3	0.000
	Three‐segment	33.5 ± 12.4	41.5 ± 12.5	0.000
	Total	24.4 ± 10.6	29.3 ± 11.1	0.000
Intraoperative fluoroscopy (*n*)	Single‐segment	26.3 ± 7.5	33.4 ± 8.3	0.000
	Double‐segment	37.7 ± 8.5	46.7 ± 10.3	0.000
	Three‐segment	48.7 ± 16.2	60.2 ± 19.5	0.000
	Total	30.2 ± 14.3	38.2 ± 15.6	0.000
Estimate blood loss (mL)		101.6 ± 62.9	105.5 ± 66.3	0.426
Hospital stay (days)		3.95 ± 0.81	3.85 ± 0.77	0.238

### 3.3 Radiographic Assessment

**FIGURE 6:**
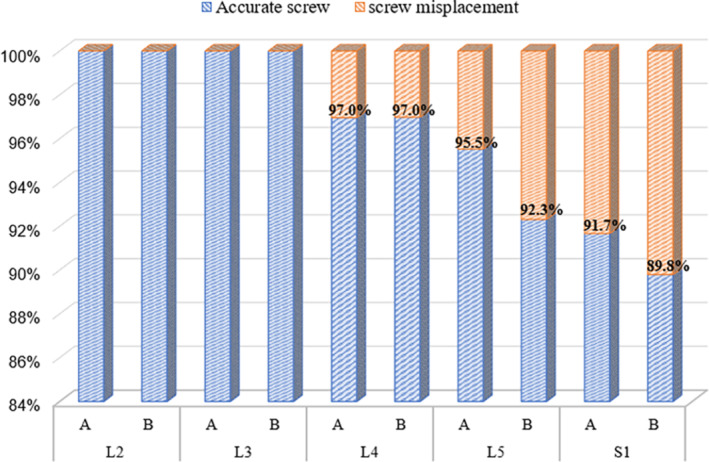
Clinically acceptable screws in different segments between two groups.

Overall, the proportion of clinically acceptable screws was 95.9% in TDA group and 94.9% in PPSF group. The Chi‐square test showed no significant difference between the two groups (*χ* ^2^ = 1.206, *p* = 0.272) (Table 3). However, among different segments, the clinically acceptable screws at L5 and S1 levels in TDA group were higher than those in PPSF group (Figure 6).

**TABLE 3:** Accuracy of pedicle screw placement between the two groups.

Grade	No. of screws (%)		*p*
	Group A	Group B	
A	908 (83.8%)	833 (82.6%)	0.491
B	132 (12.1%)	124 (12.3%)	0.931
A + B	1040 (95.9%)	957 (94.9%)	0.272
C	41 (3.8%)	47 (4.7%)	0.316
D	3 (0.3%)	4 (0.4%)	0.634
C + D	44 (4.1%)	51 (5.1%)	0.272
Total	1084	1008	—

In addition, the lateral screw misplacement in TDA group was higher than that in PPSF group (5.9% vs. 2.8%, *p* < 0.05), the medical screw misplacement in TDA group was lower (1.5% vs. 7.3%, *p* < 0.05) (Table 4).

**TABLE 4:** Medial and lateral distribution of misplaced screws.

		Misplaced screws	Accuracy screws	Total	Misplacement rate	*p*
Medial	Group A	8	534	542	1.5%	0.000
	Group B	37	467	504	7.3%	
Lateral	Group A	32	510	542	5.9%	0.014
	Group B	14	490	504	2.8%	

In TDA group, facet joint invasion of Grade 0, Grade 1, Grade 2, and Grade 3 were 82.4% (893/1084), 12.0% (130/1084), 4.0% (44/1084), and 1.6% (17/1084), respectively, and the average FJV was 0.25 ± 0.6. In PPSF group, FJV of Grade 0, 1, 2, and 3 accounted for 64.4% (649/1008), 23.2% (234/1008), 8.7% (88/1008), and 3.7% (37/1008), respectively, and the average FJV was 0.52 ± 0.8, and the difference was statistically significant (*p* < 0.05) (Table 5).

**TABLE 5:** Grade of facet joint violation between the two groups.

FJV	Group A	Group B	*p*
Grade 0	893 (82.4%)	649 (64.4%)	0.000
Grade 1	130 (12.0%)	234 (23.2%)	0.000
Grade 2	44 (4.0%)	88 (8.7%)	0.000
Grade 3	17 (1.6%)	37 (3.7%)	0.002
Mean FJV grade	0.25 ± 0.6	0.52 ± 0.8	0.000

### 3.4 Clinical Outcomes

In both TDA group and PPSF group, postoperative back and leg pain and the JOA score were significantly improved (*p* < 0.05). However, there were no significant differences in the pre‐ and postoperative VAS score for back and leg pain and the JOA score between the two groups (*p* > 0.05). Moreover, there were no significant differences in JOA recovery rate, intervertebral fusion rate, and complications rate (*p* > 0.05) (Table 6). Complications in TDA group included three cases of transient radicular pain, two cases of internal fixation loosening, one case of dural injury, and one case of incision infection. Meanwhile, complications in PPSF group included three cases of transient radicular pain, two cases of internal fixation loosening, two cases of dural injury, and one case of incision infection. After treatment, all patients were satisfied with the clinical outcomes at their last follow‐up visit.

**TABLE 6:** Comparison of clinical outcomes between the two groups.

		Group A	Group B	*p*
VAS for LBP	Preoperative	5.4 ± 2.2	5.6 ± 1.9	0.67
	2‐year follow‐up	0.8 ± 0.6*	0.8 ± 0.7*	0.88
VAS for leg pain	Preoperative	7.8 ± 1.0	7.7 ± 0.9	0.68
	2‐year follow‐up	2.9 ± 0.9*	2.7 ± 1.2*	0.49
JOA score	Preoperative	14.2 ± 1.7	13.9 ± 2.0	0.48
	2‐year follow‐up	24.1 ± 1.6*	23.7 ± 1.7*	0.60
JOA recovery rate (%)		78.7 ± 6.6	77.0 ± 3.9	0.11
Intervertebral fusion rate (%)		90.7	89.9	0.53
Complications		7/231	8/214	0.68

* Statistically significant difference between the pre‐ and postoperative clinical outcomes.

## 4 Discussion

### 4.1 Feasibility of Trajectory Dynamic Adjustment Technique

Previous studies have demonstrated that PPSF technique has the advantages of small trauma, rapid postoperative recovery, and satisfactory surgical effects [3, 18, 19, 20, 21]. However, PPSF also has some potential limitations. The biggest drawback of this technique is its fluoroscopic dependence, which will inevitably increase the radiation exposure of doctors and patients [22]. In the process of percutaneous puncture, the needle tip must reach the posterior edge of the vertebral body in the lateral view when it reaches the inner wall in the anteroposterior view. This is the fundamental principle of preventing nerve injury by ensuring the needle does not enter the spinal canal [23]. Practitioners should always remember this “inner wall protection principle.” For the lower lumbar spine with large pedicle, it is safer for the needle to be slightly offset to the outer side compared to the inner side. In the past, practitioners often struggled with the incorrect idea that the puncture entry point should be within the pedicle projection. Therefore, they spend a lot of time adjusting the entry point to positions like 3 o'clock or 9 o'clock on the pedicle, which are often on inclined or vertical surfaces, making stable anchoring difficult to achieve. In the present study, we proposed a modified PPSF technique, trajectory dynamic adjustment technique. In this modified technique, the entry point of the needle is slightly offset 3–5 mm to the outer side, directly facing the base of the transverse process, and relatively horizontal, making it easier to achieve stable bony anchoring. Subsequently, based on the initial position of the entry point, the needle tip is gradually adjusted to the center of the pedicle while the needle advances. The feasibility of this technique is due to the self‐centralization of the screw during insertion, that is, even if the final position of the needle tip is close to the inferior wall of the pedicle, the thick screw will automatically move to the soft cephalic side of the pedicle when it meets the hard cortical bone of the pedicle during the screw insertion.

The greatest advantage of the TDA technique is that it is easier to achieve good initial anchoring of the needle because the entry point is relatively flatter instead of on steep slopes. Another major advantage is that TDA technique reduces the requirement for the entry point position, saving time on repeatedly adjusting the entry point.

The results of this study confirmed that the insertion time of the needle in TDA group was significantly lower than that in PPSF group (24.4 ± 10.6 vs. 29.3 ± 11.1, *p* < 0.05), and the fluoroscopy frequency in TDA group was also significantly lower than that in PPSF group (30.2 ± 14.3 vs. 38.2 ± 15.6, *p* < 0.05). At the same time, with the increase of surgical level, the TDA technique has more advantages in terms of needle insertion time and radiation exposure.

### 4.2 The Accuracy of Screw Placement

The accuracy of pedicle screw placement is crucial in spinal surgery. Previous studies have shown that the accuracy of pedicle screw placement with traditional PPSF is not consistent, ranging from 60% to 98% [8, 24, 25, 26]. The anatomy of the pedicle is special, which constitutes the lateral wall of the spinal canal and is adjacent to the dural sac and nerve roots. Therefore, it is very dangerous to penetrate the inner wall of the pedicle during operation. The accurate placement of pedicle screws requires the accumulation of surgical experience, especially for PPSF technique, because it lacks the gross visualization and tactile feedback compared with open surgery. In the present study, the accuracy of screw placement was 95.9% (1040/1084) in TDA group and 94.9% (957/1008) in PPSF group. Both groups had high accuracy of screw placement, and the difference was not statistically significant (*p* > 0.05). However, in different segments, the clinically acceptable screws at L5 and S1 levels in TDA group were higher than those in PPSF group (95.5% vs. 92.3%, 91.7% vs. 89.8%, respectively). Considering that there were larger pedicle angulation and more facet joint hyperplasia in L5 and S1 segments [27, 28], a more lateral tilt angle of the needle insertion can solve this problem to a certain extent. Further analysis of the medial and lateral distribution of misplaced screws revealed that the TDA group was more prone to screws protruding the outer wall of the pedicle than PPSF group (5.9% vs. 2.8%, *p* < 0.05), which generally does not cause nerve stimulation. Conversely, the PPSF group was more prone to screws penetrating the inner wall of the pedicle (7.3% vs. 1.5%, *p* < 0.05), causing stimulation to the nerve root or dural sac and resulting in neurological symptoms. As a result, TDA technique exhibited a higher level of neurological safety.

### 4.3 Facet Joint Violation

It has shown that PPSF has a higher FJV rate than open surgery, which can reach 22.4%–58% [15, 29, 30]. Considering that the facet joint does not have a typical fluoroscopic projection, sometimes it overlaps with the pedicle projection. Surgeons often adhere to the erroneous idea that the entry point should be within the projection of the pedicle, which can lead to the entry point being located on the superior facet joint. The higher rate of superior facet joint violation is a result of this misplaced entry point. Additionally, violation of the facet joint caused by pedicle screws is a risk factor for adjacent segment degeneration [31, 32]. The TDA technique can allow for a more lateral entry point, which can theoretically reduce the FJV. The results in this study proved that TDA technique have a lower FJV rate. Patel et al. [33] point out that PPSF has a higher FJV rate, which is related to the intraoperative fluoroscopic imaging of the facet joint on the projection of the pedicle, and the relatively lateral entry point helps to maintain an appropriate distance between the screw and the superior facet joint, which is the key to prevent the screw from violating the facet joint.

### 4.4 Clinical Outcomes

The clinical efficacy of traditional PPSF has been confirmed. It can be used alone in the treatment of thoracolumbar fractures, or combined with discectomy and interbody fusion for the treatment of spinal degenerative diseases. Luo et al. [34] conducted a meta‐analysis of 35 cohort studies and confirmed the clinical efficacy of PPSF in the treatment of thoracolumbar fractures. Kotani et al. [35] used PPSF combined with posterolateral interbody fusion to treat 80 patients with degenerative lumbar spondylolisthesis and spinal canal stenosis, and were followed up for 2 years, with satisfactory results. In this study, the postoperative VAS score and JOA score of the TDA group and PPSF group were significantly improved compared with those before the operation. In addition, there was no significant difference in the pre‐ and postoperative VAS score and JOA score between the two groups, and there was no significant difference in the JOA recovery rate, interbody fusion rate, and the complications rate between the two groups, which suggests that good clinical outcomes can be achieved in both groups.

### 4.5 Strengths and Limitations

This is the first study to propose the trajectory dynamic adjustment technique, which offers the benefits of excellent clinical efficacy in operation time, radiation exposure, facet joint invasion. Unfortunately, limitations of this study include its retrospective design, small sample size, single‐center study. It is hoped that meticulously designed multicenter, large sample size, prospective, randomized, controlled study will validate the results of the present study.

## 5 Conclusion

In conclusion, both PPSF and TDA technique are safe and effective procedures in the treatment of lumbar degenerative disease, which have similar screw placement accuracy and clinical outcomes. However, TDA technique has shorter surgical time, lower radiation exposure, and lower facet joint violation rate compared with PPSF technique, which presents a promising option. Further research and long‐term follow‐up are needed to confirm our results.

## Author Contributions

Hao Li, Zhiguo Ding, and Bin Wei are responsible for data collection, statistical analysis and writing the main manuscript text. Zhihao Ma, Jing Xie, Yonghao Tian, Lianlei Wang, and Xinyu Liu contributed to the data collection, literature reviewing, and statistical analysis. Suomao Yuan proposed the concept and revised the article finally.

## Conflicts of Interest

The authors declare no conflicts of interest.

## Data Availability

The authors has nothing to report.
